# Geographic atrophy progression and subretinal drusenoid deposits in a real-world Japanese cohort

**DOI:** 10.1007/s10384-026-01388-3

**Published:** 2026-06-29

**Authors:** Takuya Takayama, Hidenori Takahashi, Taiki Tsuge, Mayumi Chiba, Marieh Esmaeelpour, Satoru Inoda, Hironobu Tampo, Yuto Hashimoto, Kyouta Kimura, Yusuke Fukuju, Toshikatsu Kaburaki, Yasuo Yanagi

**Affiliations:** 1 Department of Ophthalmology, Jichi Medical University, Shimotsuke, Tochigi Japan; 2 Center for Cyber Medicine Research, University of Tsukuba, 1-1-1 Tennodai, Tsukuba, Ibaraki 305-8575 Japan; 3 DeepEyeVision Inc, Shimotsuke, Tochigi Japan; 4 Boehringer Ingelheim International GmbH, Ingelheim am Rhein, Germany; 5 Nippon Boehringer Ingelheim Co., Ltd., Tokyo, Japan; 6 Department of Visual Reconstructive Surgery, Yokohama City University, Yokohama, Kanagawa Japan; 7 Retina Research Group, Singapore Eye Research Institute, Singapore, Singapore

**Keywords:** Geographic atrophy, Japanese, Subretinal drusenoid deposits, Fundus autofluorescence pattern subtypes

## Abstract

**Purpose:**

To investigate the natural course of geographic atrophy (GA) in Japanese patients, with a focus on factors associated with the change of GA size over time and visual acuity decline.

**Study design:**

Retrospective, single-center observational study.

**Methods:**

We reviewed 40 eyes of 40 patients with GA and longitudinal fundus autofluorescence (FAF) imaging. Annual progression rates were calculated as square root–transformed (mm/year). Assessed factors included age, sex, smoking history, diabetes, hypertension, baseline best-corrected visual acuity (BCVA), baseline lesion size, foveal center involvement, choroidal thickness, FAF subtypes, and presence of subretinal drusenoid deposits (SDD). Visual outcomes were assessed as change in BCVA (ΔBCVA, logarithm of the minimum angle of resolution (logMAR)).

**Results:**

Median age was 75.5 years; 50% of eyes had SDD. The median annual GA progression rate was 0.16 mm/year in square root units. Baseline GA area and the presence of SDD were independent predictors of GA progression (β = −0.0837, p = 0.015; β = +0.3064, p = 0.009, respectively). In multivariable analysis, baseline GA area also remained a significant predictor of visual deterioration (β = +0.1369, p = 0.002). When stratified by FAF patterns, there was significant worsening of BCVA in the Diffuse (p = 0.0027) and Banded (p = 0.0117) subtypes, whereas the None and Other subtypes did not show significant changes in BCVA over one year.

**Conclusions:**

SDD was identified as a significant risk factor for GA progression in Japanese patients. These findings improve understanding of natural history of GA in Japanese population and may inform risk stratification.

**Supplementary Information:**

The online version contains supplementary material available at https://doi.org/10.1007/s10384-026-01388-3.

## Introduction

Age-related macular degeneration (AMD) affects the macula, the central region of the retina essential for vision, causing visual impairment and representing a major cause of blindness in developed countries [1]. With the rapidly aging population, the number of AMD patients is projected to reach 288 million by 2040, creating substantial healthcare and socioeconomic burdens [2].

Advanced AMD can be classified into two forms: neovascular AMD, characterized by choroidal neovascularization, and atrophic AMD, involving retinal pigment epithelium (RPE) atrophy, also known as geographic atrophy (GA) [3]. GA is a complex and heterogenous disease and exhibits ethnic differences, with a lower prevalence reported in Asian populations compared to Caucasians [4, 5]. While extensive data on the prevalence, risk factors, and clinical characteristics of GA have been accumulated in Caucasian populations [6], data from Asian populations remain limited. Reflecting its heterogeneity of underlying pathophysiology, progression rates and precursor lesions which differs across different populations. Specifically, Asian, including Japanese populations are reportedly characterized by lower prevalence and notably slower progression rates compared to non-Asian populations. Reported Japanese GA features include male predominance, with lesions often smaller and less bilateral. The higher frequency of pachychoroid GA and smaller lesion sizes may underlie the differences in disease progression. However, dedicated data in the Japanese population remain limited.

In recent years, therapeutic approaches targeting the complement system have emerged. Pegcetacoplan, a peptide drug targeting the complement system (particularly C3), was approved by the US FDA in 2023 as the first treatment for GA, demonstrating up to a 21% reduction in GA progression in the pivotal OAKS and DERBY phase 3 trials [7]. However, these trials included very few Asian participants, leaving the efficacy and safety in Asian populations insufficiently characterized. Avacincaptad pegol, another complement inhibitor targeting C5, received conditional approval for the treatment of GA secondary to atrophic AMD in Japan [8, 9]. This therapy has not yet been widely introduced into clinical practice, and real-world evidence in Asian populations remains scarce.

Building upon these considerations, the present study investigated factors associated with the progression of GA and longitudinal changes in visual acuity among Japanese patients with GA. By characterizing these associations, we aimed to enhance understanding of the natural history of GA in Asian populations and to provide essential information to optimize the patient selection for the administration of complement inhibitors.

## Materials and methods

### Ethics statement

This study was conducted in accordance with the tenets of the Declaration of Helsinki and was approved by the Ethics Committee of Jichi Medical University Hospital (Approval No. CU 24-018). Participants were provided with a publicly available information document and received an oral explanation of the study. Verbal informed consent was obtained, and only de-identified data were used for analysis.

### Study participants

This retrospective, single-center study included consecutive patients diagnosed with GA associated with AMD at the Department of Ophthalmology, Jichi Medical University Hospital, between January 2014 and December 2023.

Patients were eligible if they had at least two longitudinal fundus autofluorescence (FAF) images acquired ≥3 months apart using a confocal scanning laser ophthalmoscope. Eyes were excluded if they had inherited retinal diseases, high myopia, chronic central serous chorioretinopathy, traumatic injury, a history of RPE tear or laser photocoagulation, or evidence of macular neovascularization (MNV). All eligible patients who presented to our hospital during the study period were included, regardless of the timing of GA onset.

For patients with bilateral GA, the eye with the larger atrophic area fully captured within the imaging frame was included. Demographic and clinical information—including age, sex, smoking history, systemic comorbidities (diabetes and hypertension), and best-corrected visual acuity (BCVA, logarithm of the minimum angle of resolution (logMAR))—was extracted from medical records.

### Diagnosis of geographic atrophy and imaging modalities

Diagnosis of GA was based on multimodal imaging. FAF served as the primary imaging modality for identifying atrophic areas, determining lesion borders, and quantifying lesion size. GA was first detected on FAF based on hypoautofluorescent areas with sharply demarcated borders. To ensure diagnostic accuracy and avoid misinterpretation from media opacities or artifacts, FAF findings were cross-referenced with:

- Color fundus photography (CFP) for pigmentary abnormalities and fundus-level confirmation
- Near-infrared reflectance (NIR) for delineation of RPE atrophy patterns
- Structural optical coherence tomography (OCT) for verifying outer retinal atrophy

The three criteria of the Classification of Atrophy Meeting (CAM) Report 3 [10] were confirmed using structural OCT:

1. a region of hypertransmission ≥250 µm;
2. attenuation or disruption of the RPE ≥250 µm;
3. overlying photoreceptor degeneration without evidence of scrolled RPE or RPE tear.

OCT was also used to confirm the presence or absence of drusen and subretinal drusenoid deposits (SDD).

### Choroidal thickness measurement

Choroidal thickness was measured using structural OCT images. Choroidal thickness was defined as the vertical distance from the outer border of the retinal pigment epithelium to the choroid–scleral interface at the foveal center. Measurements were performed using the built-in caliper tool of the OCT device.

### Image acquisition, segmentation, and annotation

**Fig. 1:**
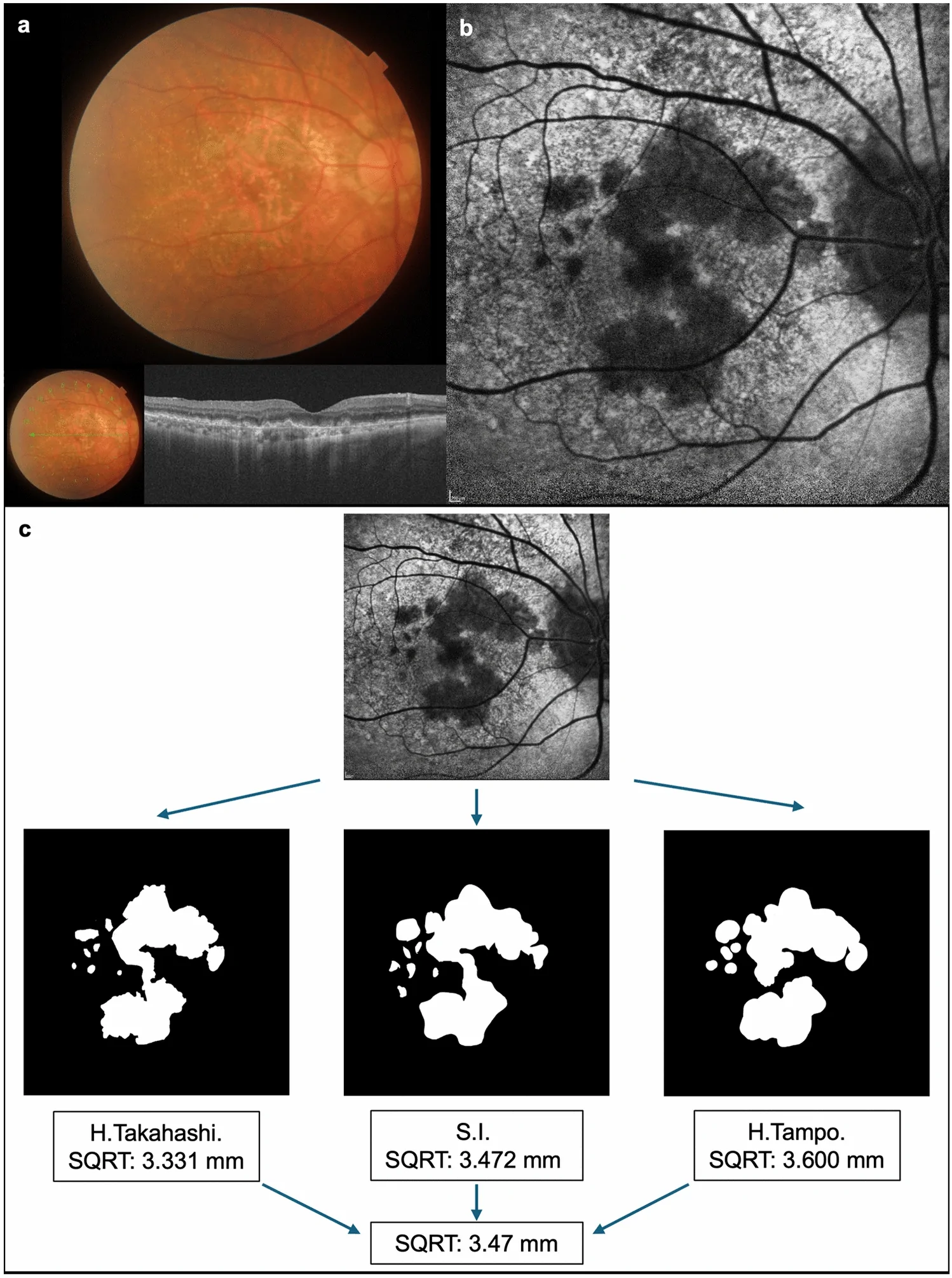
Pixel-level annotation of geographic atrophy (GA) in the right eye of a representative patient with GA. The patient was an 80-year-old woman with a best-corrected visual acuity (BCVA) of 0.10 logarithm of the minimum angle of resolution (logMAR). **a** Color fundus photograph and optical coherence tomography (OCT) demonstrating the presence of GA. **b** Fundus autofluorescence (FAF) image showing a well-demarcated hypoautofluorescent area consistent with the GA lesion. The FAF pattern subtype was classified as diffuse type. **c** Annotation of GA performed on the FAF image. The annotated regions were highlighted in white and were independently delineated by three graders: two medical retina specialists (H.Takahashi. and S.I.) and one orthoptist (H.Tampo.) with expertise in ophthalmic imaging. The GA area was measured using image analysis software (MENOU-TE; MENOU, Japan), yielding a measured area of a Square root–transformed (SQRT) 3.47 mm

FAF images were acquired using the Heidelberg Retina Angiograph (HRA2; Heidelberg Engineering). Lesion segmentation and area measurements were performed using dedicated image-analysis software (MENOU-TE, MENOU). Segmentation was performed primarily on FAF, with CFP and NIR used as topographic references to refine lesion boundaries. OCT was used as a structural reference to avoid segmentation of non-atrophic hypofluorescence and to confirm RPE loss. Image annotation and lesion segmentation were independently performed by three graders—two medical retina specialists (H. Takahashi and S. I.) and one orthoptist (H. Tampo). Disagreements were resolved through consensus, and the mean value of the three independent measurements was used in the analysis (Fig. 1).

### Foveal center involvement

Foveal center involvement at baseline was evaluated using FAF images with reference to OCT. Three independent graders assessed whether the GA lesion involved the foveal center. In cases of disagreement, the final classification was determined by majority vote among the three graders.

### FAF pattern subtype classification

FAF patterns were categorized into five subtypes according to the classification system proposed by the Fundus Autofluorescence in Age-related Macular Degeneration (FAM) study group [11]:

- None: No evident increase in FAF around the atrophic border.
- Focal: Characterized by single or individual small spots of markedly increased FAF (diameter <200 µm) located near the junctional zone.
- Banded: A continuous ring or band of increased FAF surrounding the GA lesion.
- Diffuse: Widespread increased FAF extending beyond the GA margin, not limited to the border.
- Other: Patterns not fitting into the above four categories.

Classification was performed primarily using FAF, with OCT used as needed to differentiate drusen-related changes from true FAF abnormalities. FAF pattern classification was independently performed by three graders. In cases of disagreement, the final classification was determined by consensus through joint review and discussion among the graders.

### Outcomes

The primary outcome was to identify factors associated with the progression of GA. Annual GA progression rates were calculated using square root–transformed (SQRT) rate (mm/year): change in the square root of GA area between baseline and final visit divided by follow-up years, to reduce baseline size dependence [12].

Assessed factors included age, sex, smoking history, systemic comorbidities (diabetes and hypertension), baseline BCVA, baseline GA area (SQRT), the presence of SDD, foveal center involvement, choroidal thickness, and FAF pattern subtypes.

The secondary outcome was to identify factors associated with change in visual acuity (ΔBCVA, logMAR) during follow-up. The same assessed factors as in the primary outcome were evaluated.

### Statistical analysis and figure generation

#### Primary outcome: GA progression

- Univariate analysis
  For the primary outcome of annual GA progression, univariate associations were evaluated using Welch’s t-test for two-group comparisons (e.g., presence vs absence of SDD) and Spearman’s rank correlation for continuous predictors. Comparisons across the five FAF pattern subtypes were performed using an overall ANOVA.
- Multivariable analysis
  To identify independent factors associated with GA progression, multivariable linear regression analysis was performed. Age and sex were forced into the model based on clinical relevance. For all other candidate variables, stepwise variable selection guided by the Akaike information criterion (AIC) was conducted. Variables were iteratively added or removed to minimize AIC, and the model with the lowest values was selected as the final model. Candidate predictors included smoking history, diabetes, hypertension, baseline BCVA, baseline GA area (SQRT), lens status, axial length, presence of SDD, foveal center involvement, choroidal thickness, and FAF pattern subtype.

#### Other outcome: visual acuity change (ΔBCVA)

- Univariate analysis
  For the secondary outcome, differences in ΔBCVA across the five FAF pattern subtypes were analyzed using an overall ANOVA. Within-group comparisons of baseline versus final BCVA for each FAF subtype were assessed using the Wilcoxon signed-rank test. As a supplementary analysis for the distributional comparison, the Kruskal–Wallis test and Dunn–Bonferroni post-hoc correction were applied.
- Multivariable analysis
  Multivariable linear regression was performed to evaluate independent predictors of ΔBCVA. Stepwise variable selection based on AIC was used to determine the final model. Candidate predictors included age, sex, smoking status, diabetes, hypertension, baseline BCVA, baseline GA area (SQRT), lens status, axial length, presence of SDD, foveal center involvement, choroidal thickness, and FAF pattern subtype.
  All statistical analyses and figures were generated using Python (version 3.10; Python Software Foundation).

## Results

### Patient characteristics

A total of 40 eyes from 40 patients with GA were included (Table 1). The median age was 75.5 years (range, 60–92), with an equal distribution of males and females (20 patients each). Ten patients were never smokers, 12 were ever smokers, and smoking history was unknown in 18 patients. Four patients had diabetes, and 20 had hypertension. The median baseline logMAR BCVA was 0.15 (0.08–1.15). SDD were present in 20 eyes. The median baseline GA area was 2.3 mm (0.5–7.8). The median follow-up duration was 17.9 months (3.0–125.2). The distribution of follow-up duration is shown in Supplementary Fig. S1. The median annual GA progression rate was 0.16 mm/year (-0.71-1.10).

**Table 1:** Participant characteristics at baseline

Characteristic	Value
Patients, n	40
Age (median, range), yrs	75.5, 60–92
Sex, n, male/female	20 (50%)/20 (50%)
Smoking history, n, never/ever/unknown	10 (25%)/12 (30%)/18 (45%)
Diabetes, n, yes/no	4 (10%)/36 (90%)
Hypertension, n, yes/no	20 (50%)/20 (50%)
Baseline BCVA, logMAR (median, range)	0.15, −0.08–1.15
Fellow eye BCVA, logMAR (median, range)	0.52, −0.08–2.00
Baseline GA area (SQRT) (median, range), mm	2.3, 0.5–7.8
Laterality, n, unilateral/bilateral	10 (25%)/30 (75%)
Lens status, n, phakic/IOL	20 (50%)/20 (50%)
Axial length (median, range), mm	23.39, 20.58–25.85
SDD, n, present/absent	20 (50%)/20 (50%)
Foveal center involvement, n, yes/no	19 (47.5%)/21 (52.5%)
Choroidal thickness, (median, range), μm	125.0, 16.0–581.0
Follow-up duration (median, range), months	17.9, 3.0–125.2
Annual GA progression (median, range), mm/year	0.16, −0.71–1.10

*n* number of patients; *BCVA* best-corrected visual acuity; *logMAR* logarithm of the minimum angle of resolution; *GA* geographic atrophy; *IOL* intra ocular lens; *SDD* subretinal drusenoid deposits.

**Fig. 2:**
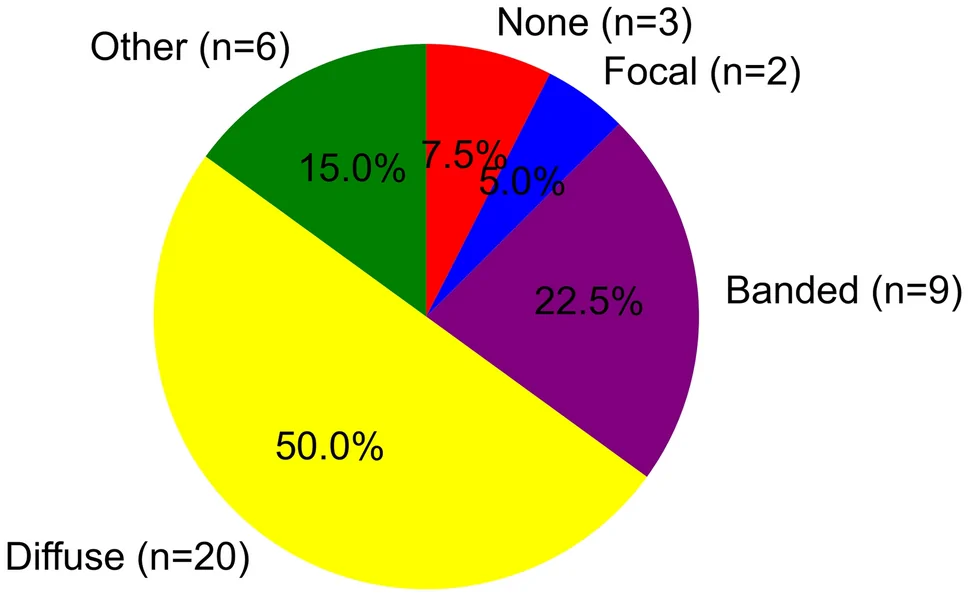
Distribution of FAF Pattern Subtypes in Jichi Medical University. The diffuse type was the most frequently observed, followed by the banded, other, none, and focal

Figure 2 illustrated the distribution of FAF pattern subtypes. None was observed in 3 eyes, focal in 2 eyes, banded in 9 eyes, diffuse in 20 eyes, and other in 6 eyes, with the diffuse subtype observed most frequently.

### Intergrader reliability of GA area and SDD assessments

Intergrader agreement for GA area (SQRT) measurements was excellent (ICC = 0.991). Bland–Altman analyses demonstrated minimal systematic bias among three graders, with narrow 95% limits of agreement (Supplementary Fig. S2). Intergrader agreement for the presence of SDD was perfect (Fleiss’ κ = 1.00).

### Factors associated with GA progression

Univariate analyses showed that the presence of SDD was significantly associated with faster GA progression (p = 0.037). Other variables, including age, sex, smoking history, diabetes, hypertension, baseline BCVA, baseline GA area (SQRT), lens status, axial length, foveal center involvement, choroidal thickness, and FAF pattern subtypes, were not significantly associated with GA progression (Table 2).

**Table 2:** Univariate analysis of factors associated with GA progression (mm/year)

Predictor	Category/Unit	n	Mean or β	95% CI	*p* value
Age	per 1 year	40	β = −0.0014	−0.0165 to 0.0138	0.857
Sex	Female	20	0.260	0.056 to 0.464	
Sex	Male	20	0.196	0.055 to 0.336	0.590
Smoking history	Never	10	0.422	0.218 to 0.626	
Smoking history	Ever	12	0.153	−0.010 to 0.316	
Smoking history	Unknown	18	0.170	−0.053 to 0.393	
Smoking history	Overall ANOVA	–	–	–	0.161
Diabetes	No	36	0.246	0.116 to 0.375	
Diabetes	Yes	4	0.070	−0.233 to 0.372	0.174
Hypertension	No	20	0.195	0.006 to 0.384	
Hypertension	Yes	20	0.261	0.101 to 0.421	0.582
Baseline BCVA	per 1 logMAR	40	β = 0.0123	−0.3024 to 0.3269	0.938
Baseline GA area (SQRT)	per 1 mm	40	β = −0.0518	−0.1180 to 0.0143	0.121
Lens status	Phakic	20	0.234	0.110 to 0.358	
Lens status	IOL	20	0.222	0.006 to 0.438	0.920
Axial length	per 1 mm	40	β = −0.0196	−0.1378 to 0.0987	0.740
SDD	Absent	20	0.106	−0.055 to 0.268	
SDD	Present	20	0.350	0.179 to 0.520	**0.037**
Foveal center involvement	No	21	0.275	0.092 to 0.458	
Foveal center involvement	Yes	19	0.176	0.013 to 0.338	0.400
Choroidal thickness	per 1 μm	40	β = −0.0006	−0.0016 to 0.0005	0.283
FAF pattern	Banded	9	0.119	−0.050 to 0.288	
FAF pattern	Diffuse	20	0.253	0.037 to 0.470	
FAF pattern	Focal	2	0.384	−3.200 to 3.968	
FAF pattern	None	3	0.433	−0.608 to 1.473	
FAF pattern	Other	6	0.152	0.018 to 0.286	
FAF pattern	Overall ANOVA	–	–	–	0.686

Annual GA progression was evaluated using univariate analyses for clinical background factors including age, sex, smoking, diabetes, and hypertension, as well as ocular factors including baseline BCVA, baseline GA area (SQRT), lens status, axial length, foveal center involvement, choroidal thickness. In addition, the associations with the presence of SDD and each FAF pattern subtype (five categories) were assessed. Mean values and 95% confidence intervals are shown for each factor. *P* values were calculated using linear regression for continuous variables and Welch’s t-tests or ANOVA for categorical variables. SDD demonstrated a statistically significant association with GA progression. Bold *P* values indicate statistical significance (*P* < 0.05)

Stepwise selection with age/sex forced into the model identified four variables (age, sex, baseline GA area (SQRT), and SDD) as the optimal multivariable model (lowest AIC). Baseline GA area (SQRT) (β = −0.0837, p = 0.015) and SDD (β = +0.3064, p = 0.009) were selected as significant independent predictors of GA progression (Table3).

**Table 3:** Multivariate analysis of factors associated with GA progression (mm/year)

Predictor	β	95% CI	*p* value
Age	−0.0081	−0.022 to 0.006	0.261
Sex	−0.1573	−0.392 to 0.078	0.183
Baseline GA area (SQRT)	−0.0837	−0.150 to -0.017	**0.015**
SDD	+0.3064	0.082 to 0.531	**0.009**

Multivariate linear regression analysis was performed using stepwise variable selection. The stepwise procedure identified four variables (age, sex, baseline GA area (SQRT), and SDD) as the optimal multivariable model according to the lowest Akaike information criterion (AIC). Among these, baseline GA area (SQRT) and the presence of SDD remained statistically significant independent predictors of GA progression. Bold *P* values indicate statistical significance (*P* < 0.05)

**Fig. 3:**
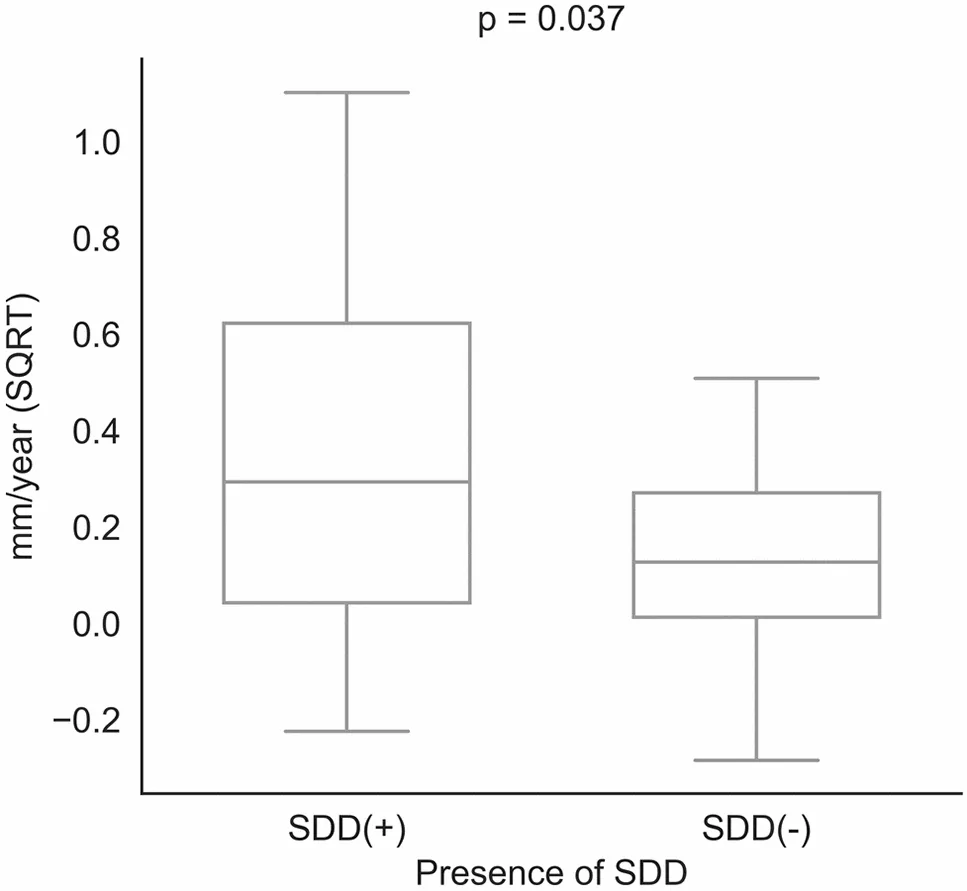
GA progression according to the presence of subretinal drusenoid deposits (SDD). Boxplots showing the annual progression rates of GA in eyes with and without SDD. Patients with SDD showed significantly faster GA progression (p = 0.037; Welch’s t-test)

Figure 3 illustrates the distribution of GA progression rates according to the presence or absence of SDD, showing consistently higher progression in eyes with SDD (p = 0.037, SQRT mm/year).

### Factors associated with BCVA changes

Univariate analysis showed that larger baseline GA area (SQRT) was significantly associated with greater visual acuity decline (ΔBCVA, logMAR; p = 0.002). FAF pattern subtypes were also significantly associated with ΔBCVA (overall ANOVA, p = 0.044). Other factors—including age, sex, smoking history, diabetes, hypertension, baseline BCVA, lens status, axial length, the presence of SDD, foveal center involvement, and choroidal thickness—were not significantly related to visual acuity change (Table 4).

**Table 4:** Univariate analysis of factors associated with change in BCVA (ΔBCVA, logMAR)

Predictor	Category/Unit	n	Mean or β	95% CI	*p* value
Age	per 1 year	40	β = −0.0035	−0.0228 to 0.0159	0.718
Sex	Female	20	0.394	0.161 to 0.627	
Sex	Male	20	0.199	−0.007 to 0.406	0.199
Smoking history	Never	10	0.420	0.030 to 0.810	
Smoking history	Ever	12	0.310	−0.030 to 0.650	
Smoking history	Unknown	18	0.219	0.021 to 0.418	
Smoking history	Overall ANOVA	–	–	–	0.571
Diabetes	No	36	0.300	0.145 to 0.455	
Diabetes	Yes	4	0.267	−0.835 to 1.369	0.931
Hypertension	No	20	0.215	0.012 to 0.417	
Hypertension	Yes	20	0.379	0.140 to 0.618	0.280
Baseline BCVA	per 1 logMAR	40	β = −0.0568	−0.4589 to 0.3454	0.777
Baseline GA area (SQRT)	per 1 mm	40	β = 0.1262	0.0493 to 0.2031	**0.002**
Lens status	Phakic	20	0.248	0.021 to 0.475	
Lens status	IOL	20	0.345	0.124 to 0.566	0.525
Axial length	per 1 mm	40	β = 0.0517	−0.0989 to 0.2023	0.492
SDD	Absent	20	0.227	0.058 to 0.396	
SDD	Present	20	0.366	0.100 to 0.632	0.363
Foveal center involvement	No	21	0.251	0.032 to 0.470	
Foveal center involvement	Yes	19	0.347	0.117 to 0.577	0.530
Choroidal thickness	Per 1 μm	40	β = −0.0009	−0.0022 to 0.0005	0.208
FAF pattern	Banded	9	0.359	0.011 to 0.708	
FAF pattern	Diffuse	20	0.338	0.114 to 0.561	
FAF pattern	Focal	2	1.040	−6.107 to 8.186	
FAF pattern	None	3	−0.059	−0.311 to 0.194	
FAF pattern	Other	6	−0.005	−0.132 to 0.122	
FAF pattern	Overall ANOVA	–	–	–	**0.044**

Change in BCVA was evaluated using univariate analyses for clinical background factors including age, sex, smoking history, diabetes, and hypertension, as well as ocular factors including baseline BCVA, baseline GA area (SQRT), lens status, axial length, foveal center involvement, choroidal thickness. In addition, the associations with the presence of SDD and each FAF pattern subtype (five categories) were assessed. Mean values and 95% confidence intervals are shown for each factor. P values were calculated using linear regression for continuous variables and Welch’s t-tests or ANOVA for categorical variables. Baseline GA area (SQRT) demonstrated a statistically significant association with change in BCVA. Bold *P* values indicate statistical significance (*P* < 0.05)

Multivariable linear regression analysis with ΔBCVA as the dependent variable was performed using an AIC-based stepwise variable selection procedure. The stepwise procedure identified five variables (age, sex, smoking history, axial length, and baseline GA area [SQRT]) as the optimal multivariable model. In the final model, baseline GA area (SQRT) remained a statistically significant independent predictor of BCVA change (β = +0.1369 per 1 mm, 95% CI 0.053–0.220; p = 0.002), whereas the other variables were not statistically significant (Table 5).

**Table 5:** Multivariate analysis of factors associated with change in BCVA (ΔBCVA, logMAR)

Predictor	β	95% CI	*p* value
Age	−0.0070	−0.025 to 0.011	0.430
Sex	−0.0808	−0.387 to 0.226	0.596
Smoking history	−0.2627	−0.543 to 0.176	0.065
Axial length	+0.096	−0.044 to 0.235	0.172
Baseline GA area (SQRT)	+0.1369	0.053 to 0.220	**0.002**

Multivariate linear regression analysis was performed using stepwise variable selection. The stepwise procedure identified five variables (age, sex, smoking history, axial length, and baseline GA area (SQRT)) as the optimal multivariable model according to the lowest AIC. Among these, baseline GA area (SQRT) remained a statistically significant independent predictor of change in BCVA. Bold *P* values indicate statistical significance (*P* < 0.05)

### FAF pattern and BCVA changes

**Fig. 4:**
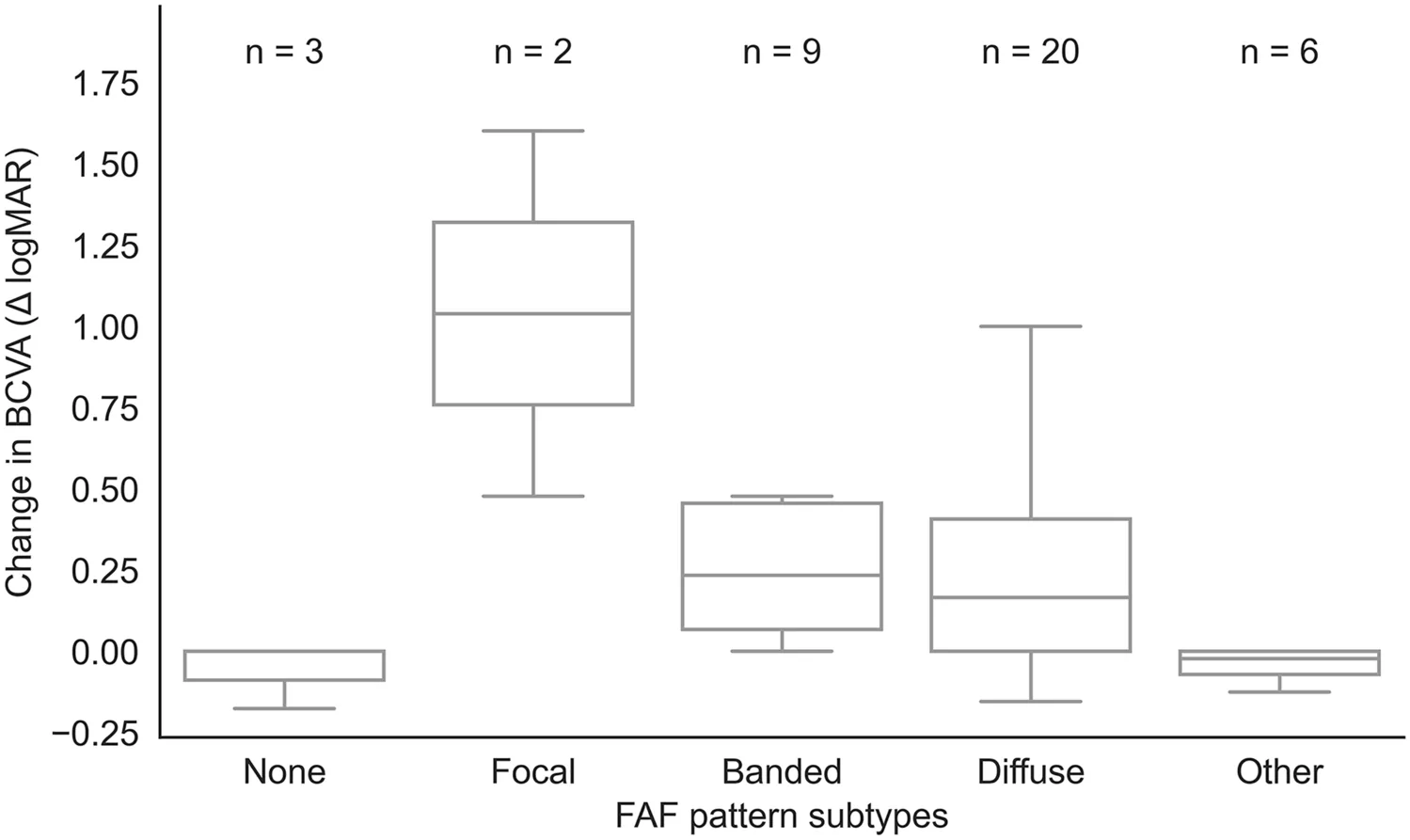
BCVA changes by FAF pattern subtypes. Boxplots show baseline and final BCVA for each FAF pattern subtype. Between-group comparisons of ΔBCVA (Final − Baseline) revealed a significant overall difference (Kruskal–Wallis, p = 0.0105), although post-hoc pairwise comparisons were not significant after Bonferroni correction

Between-group comparisons of ΔBCVA among FAF subtypes revealed an overall significant difference (Kruskal–Wallis, p = 0.0105) (Fig. 4). However, post-hoc pairwise comparisons did not remain statistically significant after Bonferroni correction. Within-group analyses showed significant worsening of BCVA in the Diffuse (p = 0.0027) and Banded (p = 0.0117) FAF pattern subtypes, whereas the None and Other subtypes did not show significant changes in BCVA (Table 6). There were only two cases with Focal subtypes.

**Table 6:** Within-group comparisons of baseline and final BCVA (logMAR) by FAF pattern subtypes

FAF pattern	n	Baseline BCVA (mean)	Final BCVA (mean)	Mean ΔBCVA (logMAR)	*P* value (Wilcoxon)
None	3	0.440	0.381	−0.059	0.3173
Focal	2	0.009	1.048	+1.040	0.5000
Banded	9	0.438	0.797	+0.359	**0.0117**
Diffuse	20	0.291	0.629	+0.338	**0.0027**
Other	6	0.449	0.444	−0.005	0.7150

Within-group comparisons of baseline and final BCVA were conducted for each FAF pattern subtype using the Wilcoxon signed-rank test. The Diffuse and Banded subtypes demonstrated statistically significant worsening of BCVA during follow-up, whereas the None and Other subtypes did not show significant changes. Bold *P* values indicate statistical significance (*P* < 0.05)

## Discussion

In this study, we investigated the natural course of GA in Japanese patients, with a particular focus on identifying factors associated with the progression of GA and changes in visual acuity. Our results demonstrate that the presence of SDD was a significant factor accelerating GA progression. Previous studies report a strong association between the presence of SDD and GA progression. Specifically, it is demonstrated that GA lesions expand more frequently in areas where SDD are present, showing a clear spatial and temporal relationship [13]. Moreover, the presence of SDD has been shown to accelerate GA progression, with particularly rapid enlargement occurring within regions containing SDD [14]. These findings establish SDD not only as a risk factor for progression to late AMD but also as a key determinant of the enlargement rate of GA lesions. Importantly, a study conducted in Japanese patients also reports that the presence of SDD was significantly associated with a greater GA progression rate [15]. Our results are consistent with these prior observations and provide further support for the role of SDD as a robust risk factor for GA progression in Japanese populations.

In our study, SDD were present in 50% of patients. This prevalence is consistent with previous reports in Japanese (38.3%–50%) [15–17] as well as in Caucasian populations (36%–37.8%) [18, 19]. These findings indicate that the frequency of SDD in Japanese patients is comparable to that observed in Western cohorts, further supporting the generalizability of SDD as a key risk factor for GA progression across different ethnic groups.

The mechanisms by which SDD contribute to GA progression have been increasingly clarified through advanced imaging studies. OCT and adaptive optics scanning laser ophthalmoscopy have revealed that regions containing SDD exhibit photoreceptor outer segment shortening and RPE degeneration, making them vulnerable to atrophy [14]. More advanced lesions show progressive RPE atrophy with outer retinal and choroidal thinning and increased light transmission [20]. Histological and imaging findings also indicate that SDD contain abundant complement components and lipid-rich material, suggesting that complement activation and impaired lipid transport contribute to photoreceptor and RPE damage [21]. Together, these structural and biochemical alterations provide a plausible mechanism by which SDD accelerate GA enlargement.

Previous studies consistently identify the diffuse and banded FAF patterns as high-risk subtypes for both GA progression and visual acuity decline, and these patterns are widely recognized internationally as important biomarkers for risk stratification and prognostic prediction in clinical practice. Large multicenter prospective studies, including those by the FAM Study Group, demonstrate that diffuse or banded patterns are strongly associated with faster lesion enlargement in non-Asian patients (mean 0.91 mm/year [95% CI 0.14–2.16]; p < 0.01), consistent with other international reports [3, 22–24]. However, this association was not observed in Asian populations, where diffuse and banded patterns did not significantly predict lesion growth [25]. In our study, diffuse and banded patterns showed a significant association with visual acuity decline, in line with previous reports. Indeed, because FAF pattern, lesion size, age, and SDD are closely related, the independent contribution of FAF pattern is difficult to disentangle in a small cohort. Taken together with previous findings, our results suggest that FAF pattern remains a clinically relevant feature in Japanese patients. Larger studies will be necessary to clarify the extent to which FAF subtypes contribute independently to visual prognosis in Asian populations.

This study has several limitations. First, it was a retrospective, single-center study with a relatively small sample size, which may have limited the statistical power. Second, there was considerable variation in follow-up duration among patients, potentially introducing bias in the estimation of progression rates. Third, although eyes with macular neovascularization and other retinal diseases were excluded, unrecognized confounding factors may still have influenced the results. In addition, differentiation between the diffuse trickling FAF pattern observed in GA secondary to AMD and GA-like phenotypes associated with inherited retinal degenerations, such as late-onset retinal degeneration (LORD) or extensive macular atrophy with pseudodrusen-like appearance (EMAP), was not systematically performed in this study. Given the phenotypic overlap among these entities on multimodal imaging—particularly in the presence of extensive pseudodrusen-like changes or SDD—misclassification cannot be completely excluded. Fourth, this study included only Japanese patients, which may limit the generalizability of the findings to other Asian populations. Fifth, because the study cohort was defined based on the availability of longitudinal FAF imaging and multiple exclusion criteria, substantial selection bias is likely present. Furthermore, this was a retrospective, hospital-based study, and prevalence estimates from such cohorts should not be interpreted as representative of the broader Japanese GA population. Referral bias, selection due to imaging availability, and unknown smoking status all limit generalizability. While the concept of pachychoroid-associated GA is still being explored, future studies will be warranted to investigate its progression characteristics once the entity is more clearly established. Sixth, a minority of eyes showed small negative GA progression values. Notably, only one eye exhibited a negative progression value, and this finding was consistent across all three graders. These negative values do not indicate true lesion regression but are most likely attributable to measurement-related factors, such as subtle differences in image quality between visits. Finally, the assessment of GA lesions was performed manually by multiple experienced graders, and measurement variability may remain despite this approach. Future large-scale, multicenter, prospective studies are needed to validate and extend the present findings.

Despite these limitations, our findings have important clinical implications. The identification of SDD as a robust risk factor for GA progression suggests that careful evaluation of SDD using multimodal imaging may be critical for predicting disease course and guiding follow-up strategies in patients with GA. Moreover, the observation that diffuse and banded FAF patterns were associated with visual acuity decline, rather than lesion growth, indicates that these subtypes may be particularly relevant for anticipating functional outcomes. Taken together, these results emphasize the importance of incorporating both SDD and FAF subtypes into clinical assessment to achieve a more comprehensive evaluation of patient prognosis.

Taken together, these findings may also have important implications for emerging complement-targeted therapies for GA. Because the efficacy of complement inhibitors has been demonstrated as a relative reduction in GA growth within each clinical trial, direct comparison of absolute progression rates (mm/year) between different populations is inappropriate, as such rates are influenced by ethnic, genetic, and environmental factors that affect the natural history of the disease. In populations with inherently slower progression, the absolute magnitude of treatment effect may appear smaller even when the relative therapeutic benefit is equivalent. Accordingly, observed differences in baseline progression should not be interpreted as reduced responsiveness to complement inhibition in Asian populations. Patients with faster progression, such as those with larger baseline lesions or the presence of SDD, may indeed experience greater absolute benefit, underscoring the importance of appropriate risk stratification in future interventional studies.

Looking forward, the emergence of new therapeutic options for GA highlights the need for reliable biomarkers that can stratify patients according to their risk of progression and visual acuity decline. Future large-scale, multicenter, prospective studies in Asian populations will be essential to validate the prognostic significance of SDD and FAF subtypes, and to determine how these factors can be integrated into clinical trial design and personalized treatment strategies.

## Supplementary Information

Below is the link to the electronic supplementary material.

Supplementary file1 (TIFF 41710 kb)

Supplementary file2 (TIFF 22749 kb)
